# Binge-Type Eating in Rats is Facilitated by Neuromedin U Receptor 2 in the Nucleus Accumbens and Ventral Tegmental Area

**DOI:** 10.3390/nu11020327

**Published:** 2019-02-02

**Authors:** Ashley E. Smith, James M. Kasper, Ara 13, Noelle C. Anastasio, Jonathan D. Hommel

**Affiliations:** 1 Center for Addiction Research, University of Texas Medical Branch, Galveston, TX 77555, USA; aesmith@utmb.edu (A.E.S.); jakasper@utmb.edu (J.M.K.); ara13c@gmail.com (Ara 13); ncanasta@utmb.edu (N.C.A.); 2 Department of Neuroscience, Cell Biology and Anatomy, University of Texas Medical Branch, Galveston, TX 77555, USA; 3 Department of Pharmacology and Toxicology, University of Texas Medical Branch, Galveston, TX 77555, USA

**Keywords:** binge-eating disorder, BED, obesity, binge-type eating, neuromedin U receptor 2, NMUR2, nucleus accumbens, ventral tegmental area

## Abstract

Binge-eating disorder (BED) is the most common eating disorder, characterized by rapid, recurrent overconsumption of highly palatable food in a short time frame. BED shares an overlapping behavioral phenotype with obesity, which is also linked to the overconsumption of highly palatable foods. The reinforcing properties of highly palatable foods are mediated by the nucleus accumbens (NAc) and the ventral tegmental area (VTA), which have been implicated in the overconsumption behavior observed in BED and obesity. A potential regulator of binge-type eating behavior is the G protein-coupled receptor neuromedin U receptor 2 (NMUR2). Previous research demonstrated that NMUR2 knockdown potentiates binge-type consumption of high-fat food. We correlated binge-type consumption across a spectrum of fat and carbohydrate mixtures with synaptosomal NMUR2 protein expression in the NAc and VTA of rats. Synaptosomal NMUR2 protein in the NAc demonstrated a strong positive correlation with binge intake of a “lower”-fat (higher carbohydrate) mixture, whereas synaptosomal NMUR2 protein in the VTA demonstrated a strong negative correlation with binge intake of an “extreme” high-fat (0% carbohydrate) mixture. Taken together, these data suggest that NMUR2 may differentially regulate binge-type eating within the NAc and the VTA.

## 1. Introduction

Binge-eating disorder (BED) is an under-recognized public health problem that is prevalent worldwide [1,2]. In the US, BED affects 3.5% of adult women and 2% of adult men, with a combined lifetime prevalence of 1.4% [1,2]. Although BED is the most common eating disorder, it remains both under-diagnosed and understudied [2]. Therefore, a more mechanistic appreciation of feeding behaviors that contribute to BED is critical to understanding disease etiology, and for identifying potential pharmacotherapeutic targets.

BED is characterized by binge episodes when an individual overconsumes highly-palatable food in a discrete period of time (≤2 h) [3,4,5]. The frequency of these binge episodes is a hallmark of BED and is used to determine the disease’s severity [3,4,5]. BED is partially attributed to the increased availability of highly palatable food, and also shares physiological, psychological, and epidemiological overlaps with obesity [1,4,6,7,8,9,10]. 

Obesity is an alarming health crisis that affects 39.8% of adults within the US alone [11]. Obese individuals present a challenging public health problem due to the increased risk of life-threatening co-morbidities [12]. Thus, the health burden of obesity is immense and demands an improved understanding of the feeding behaviors that contribute to obesity. Similar to BED, one of the feeding behaviors that contributes to obesity is the overconsumption of highly palatable food.

Highly palatable food, including high-fat food, is both energy dense and highly reinforcing in humans and rodents [13]. These reinforcing properties promote overconsumption behavior that is potentiated by dysregulation in portion control [14,15,16,17]. In fact, individuals with BED ascribe higher reinforcement value to high-fat food than individuals without BED [18]. The reinforcing properties of food are largely encoded by brain regions within the mesolimbic “reward” pathway, which includes the nucleus accumbens (NAc) and the ventral tegmental area (VTA) [19,20]. Both the NAc and the VTA have been implicated in BED and obesity [2,9,19,20,21,22,23,24,25]. Reinforcement value is mediated by the NAc—a key neuroanatomical region that regulates hedonic feeding, or food intake in the absence of a caloric deficit [9,19,20]. The VTA is also linked to reinforcement value, and affects obesogenic diet consumption [26]. 

Overall, BED and obesity share multifaceted feeding behaviors involving multiple brain circuits that regulate a complex interplay of emotions, food intake, and food reinforcement. These feeding behaviors can be probed using animal models designed for quantifying specific aspects of these diseases. Models of binge-type eating have been validated in rodents, demonstrating success in studying the overconsumption behavior characteristic of both BED and obesity [27,28,29,30,31,32,33,34,35]. The intermittent access model provides ad libitum access to regular chow and water, with limited presentation of a highly palatable food, which simulates a binge episode, similar to that observed in BED and obesity [27,29,30,32,33,35]. While this model can recreate and quantify food intake during a binge episode, it does not replicate the psychological aspects of BED, such as feelings of loss of control and feelings of shame and guilt [2,3,4,5]. Nonetheless, this model is helpful in understanding neural mechanisms underlying binge-type consumption and identifying potential regulators of this specific maladaptive feeding behavior.

Here, we investigate a novel mediator of binge-type eating, the neuropeptide receptor neuromedin U receptor 2 (NMUR2). NMUR2 is a G protein-coupled receptor predominantly expressed throughout the mammalian central nervous system. This receptor is known to regulate food intake and body weight [36,37,38,39,40]. For example, NMUR2-knockout mice are hyperphagic and demonstrate increased food intake patterns [41]. Additionally, deletion of hypothalamic NMUR2 via viral-mediated RNAi resulted in increased motivation for high-fat food and potentiated binge-type eating [32,42]. These studies indicate that NMUR2 regulates food intake—a process that is dysregulated in BED and obesity. Interestingly, the role of NMUR2 in reinforcement brain centers such as the NAc and VTA in binge-type eating has not been explored. 

Importantly, the endogenous expression of NMUR2 is highly variable among individuals and among various brain regions [41,43,44]. Neuroanatomical variability and individual differences in NMUR2 expression may underlie aspects of individual differences observed in BED and obesity [45]. The purpose of the current study was to explore relationships between synaptosomal NMUR2 expression in the NAc and the VTA—key brain regions associated with reinforcement—in a rodent model of binge-type eating across a spectrum of dietary fat and carbohydrate contents. Our findings revealed a strong positive correlation between the intake of “lower”-fat (i.e., higher carbohydrate) food and synaptosomal NMUR2 protein expression in the NAc, and a strong negative correlation between the intake of “extreme” high-fat (0% carbohydrate) food and synaptosomal NMUR2 protein expression in the VTA.

## 2. Materials and Methods 

### 2.1. Animals

Male Sprague-Dawley rats (Harlan, Houston, TX, USA) weighing 225–250 g were used in all experiments. Animals were housed individually in a temperature (i.e., 21–23 °C) and humidity (40%–50%) controlled environment with a standard 12 h light–dark cycle (lights on between 06:00 and 18:00 h). All animals were given ad libitum access to normal chow (17% fat by kcal; Teklad LM-485 Mouse/Rat Sterilizable Diet; Teklad Diets, Madison, WI, USA) and water in their home cages, including during binge sessions. Upon arrival, animals were allowed to acclimate to the room for seven days prior to handling and experimental procedures. All experiments were conducted in accordance with the NIH Guide for Use and Care of Laboratory Animals (2011), and with approval from the Institutional Animal Use and Care Committee at the University of Texas Medical Branch.

### 2.2. NMUR2 Immunohistochemistry

Rats (*n* = 4) were anesthetized with 1%–5% isoflurane, perfused with phosphate buffered saline for 5 min, followed by 4% paraformaldehyde (PFA) for 15 min. Brains were removed and sliced into 40 micronsections using a cryostat. NAc and VTA sections were stained as described in previous studies [33,43,46]. Briefly, sections were washed twice in 1× PBS, and then antigen-unmasked with 1% SDS for 5 min. Next, sections were blocked in donkey serum and incubated in 1:150 primary antibody rabbit αNMUR2 (NBP1-02351, Novus Biologicals, Littleton, CO, USA) overnight. The following day, sections were washed three times in 1X PBS, then incubated with 1:200 donkey αrabbit IgG Alexa Fluor 568 (A10042, Invitrogen, Carlsbad, CA, USA). Images were acquired using Leica True Confocal Scanner SPE in confocal mode and Leica Application Suite × software (Leica Microsystems, Wetzlar, Germany).

### 2.3. Binge Study Design

Our binge-type eating protocol was based on previously published models [27,29,32,33,35]. Briefly, male Sprague-Dawley rats (*n* = 10) were maintained on a diet of normal chow (17% fat by kcal) and water. Rats were not restricted from feeding prior to the binge period, and had ad libitum access to normal chow and water throughout the study, including during the binge period. Two days prior to experiments, animals were exposed to a mixture of 60% fat by kcal and 40% carbohydrate by kcal to minimize food neophobia. Fat–carbohydrate mixtures were prepared and weighed immediately before the binge period. Vegetable shortening (Crisco, Orrville, OH, USA) comprised of 100% fat by energy (110 kcal/12 g serving with 110 kcal from fat; 9.16 kcal/g) was mixed with marshmallow creme (Kraft, Chicago, IL, USA), containing 100% carbohydrate by energy (45 kcal/13 g serving with 0 kcal from fat; 3.46 kcal/g) to create a total of five fat mixtures of varying fat–carbohydrate content. The fat–carbohydrate mixtures were prepared as follows: 2.18 g fat and 23.12 g carbohydrate were mixed for 20% fat/80% carbohydrate/0% protein by energy, 4.36 g fat and 17.34 g carbohydrate were mixed for 40% fat/60% carbohydrate/0% protein by energy, 6.55 g fat and 11.56 g carbohydrate were mixed for 60% fat/40% carbohydrate/0% protein by energy, 8.73 g fat and 5.78 g carbohydrate were mixed for 80% fat/20% carbohydrate/0% protein by energy, and 10 g fat and 0 g carbohydrate was used for 100% fat/0% carbohydrate/0% protein by energy. One limitation is that the marshmallow creme does contain any flavoring. However, the consistency and texture of the marshmallow creme were required to promote homogeneity among the mixtures. 

Rats were randomized into five groups using a Latin square design, with each animal receiving all fat–carbohydrate mixtures in a randomized order. Each group was presented with mixtures of 20% fat (80% carbohydrate), 40% fat (60% carbohydrate), 60% fat (40% carbohydrate), 80% fat (20% carbohydrate), and 100% fat (0% carbohydrate) for a period of 5 days. Exposure to fat–carbohydrate mixtures occurred during binge 1 at the first 2-h of the dark cycle (18:00–20:00) and binge 2 at 2-h near the end of the light cycle (14:00–16:00). For all exposures, fat–carbohydrate mixtures were weighed prior to presentation and then immediately after the 2-h binge period. Differences in food weight were interpreted as total grams consumed or total calories consumed during the binge period.

### 2.4. NAc and VTA Protein Extraction

After the completion of binge studies, rats (*n* = 10) were anesthetized with 1%–5% isoflurane, and brains were removed. Both NAc and VTA were microdissected from each rat, using guidance from a rat brain atlas [26,44,47], then stored at −80 °C until protein extraction. The crude synaptosomal protein fraction was isolated as previously published to identify long-term changes in protein expression [48]. NAc and VTA tissue were homogenized in ice-cold Krebs-sucrose buffer containing protease inhibitors (P8340, Millipore Sigma, St. Louis, MO, USA) and phosphatase inhibitors (P5726, P0044 Millipore Sigma, St. Louis, MO, USA). The crude synaptosomal protein fractionation protocol enriches samples for pre- and postsynaptic proteins (i.e., presynaptic terminals, postsynaptic membranes, etc.) [49]. Samples were centrifuged for 10 min at 100 *g* at 4 °C to pellet the nuclear protein fraction. The supernatant was centrifuged for 20 min at 16,000 *g* at 4 °C to separate the crude synaptosomal protein fraction (pellet). The NAc and VTA synaptosomal protein fractions were used for all further analyses. After extraction, total protein concentration was determined using a Pierce BCA Protein Assay Kit (23225, Thermo Fisher, Waltham, MA, USA). 

### 2.5. NAc and VTA NMUR2 Protein Expression 

NAc (*n* = 10) and VTA (*n* = 10) samples were assayed for NMUR2 protein expression using the Wes^TM^ (ProteinSimple, San Jose, CA, USA) automated Western blotting system. Wes^TM^ is a capillary electrophoresis-based immunoblotting instrument, and was optimized for receptor quantification in brain tissues [48,50]. Wes™ reagents (biotinylated molecular weight marker, streptavidin-HRP fluorescent standards, luminol-S, hydrogen peroxide, sample buffer, DTT, stacking matrix, separation matrix, running buffer, wash buffer, matrix removal buffer, secondary antibodies, antibody diluent, and capillaries) were obtained from the manufacturer (ProteinSimple) and used according to the manufacturer’s specifications with minor modifications. NMUR2 polyclonal antibody (NBP1-02351, Novus Biologicals, Littleton, CO, USA) was diluted 1:50 in antibody diluent.

Samples were prepared and subjected to capillary electrophoresis-based immunoblotting as described previously [49]. Briefly, 2 µg of protein was denatured in 0.1× sample buffer and 5× master mix at 95 °C for 5 min. Then, samples and primary antibody were dispensed into a prefilled microplate (ProteinSimple). Capillary electrophoresis (375 V, 25 min, 25 °C) and immunodetection were completed using the following settings: separation matrix load, 200 s; stacking matrix load, 15 s; sample load, 9 s; antibody diluent, 30 min; primary antibody incubation, 60 min; secondary antibody incubation, 30 min; and chemiluminescent signal exposure for 15, 30, 120, 240, and 480 s. Data were analyzed using Compass Software (ProteinSimple). The Western blot analysis signal was defined as the area under the curve (AUC) for the NMUR2 peak at 48 kDa and representative “virtual blot” electrophoretic images for NMUR2 were automatically generated by the Compass Software (ProteinSimple).

### 2.6. Statistical Analysis

Binge-type eating across a spectrum of fat–carbohydrate contents in binge 1 and binge 2 was analyzed by repeated measures one-way ANOVA and Bonferroni’s multiple comparisons. All comparisons were made to the intake of a 20% fat mixture. Correlations between binge-type eating across a spectrum of fat–carbohydrate contents and NMUR2 protein expression in the NAc and the VTA were analyzed by linear regression. All statistical analyses were performed in GraphPad Prism 7.0a (GraphPad Software Inc., La Jolla, CA, USA) with an experiment-wise error rate of α = 0.05. 

## 3. Results

### 3.1. NMUR2 is Expressed Presynaptically in the NAc and the VTA

**Figure 1:**
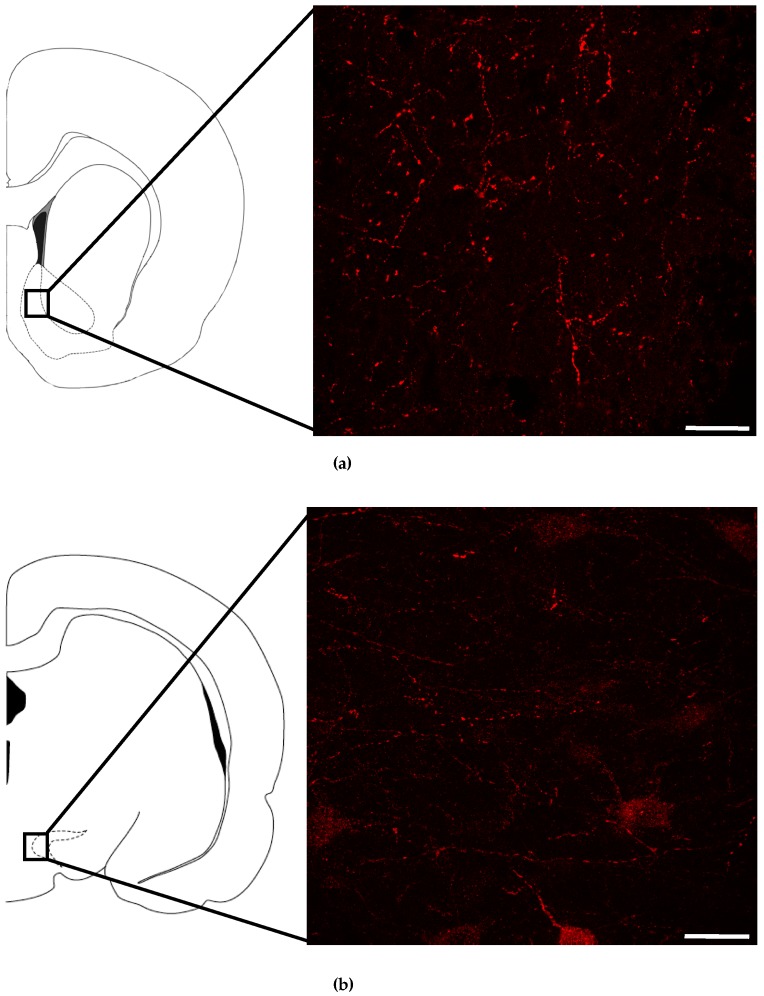
Neuropeptide receptor neuromedin U receptor 2 (NMUR2)immunostaining in the nucleus accumbens (NAc) and the ventral tegmental area (VTA). (**a**) Representative image of NMUR2 immunostaining in the NAc (bregma +1.92); (**b**) Representative image of NMUR2 immunostaining in the VTA (bregma −4.80). Images were acquired from the regions indicated at in confocal mode, scale bar = 25 µm.

Previous work demonstrated that NMUR2 is expressed postsynaptically in the hypothalamus and presynaptically in the NAc [32,46]. We confirmed NAc and VTA localization of NMUR2 with immunohistochemistry. We observed a “beads on a string” staining pattern indicative of presynaptic NMUR2 expression in the NAc and in the VTA (Figure 1).

### 3.2. Fat Content and Binge-Type Eating

**Figure 2:**
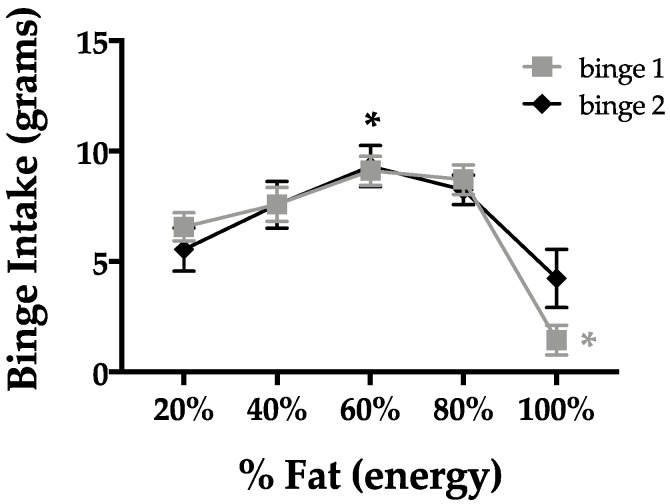
Rat binge-type eating across a spectrum of fat–carbohydrate contents. Binge-type eating in rats was highest at 60% fat (40% carbohydrate) and lowest at 80% fat (20% carbohydrate) compared to 20% fat (80% carbohydrate). * *p* < 0.05.

Using our binge-type eating paradigm, we quantified how much rats would consume during a 2-h binge period (Figure 2). Figure 2 illustrates binge intake across a spectrum of fat–carbohydrate contents during binge 1 and binge 2. Comparisons were made within each binge to the intake of a 20% fat (80% carbohydrate) mixture. In binge 1, rats consumed significantly fewer grams of the 100% fat (0% carbohydrate) mixture (*p* = 0.0003) compared to the 20% fat (80% carbohydrate) mixture (Figure 2). In binge 2, rats consumed significantly more grams of the 60% fat (40% carbohydrate) mixture (*p* = 0.010) compared to the 20% fat (80% carbohydrate) mixture (Figure 2). Notably, common Western diet foods contain percentages of fat that fall at the peak of the curve, including French fries (42% fat), potato chips (56% fat), cheesecake (63% fat), and peanut butter (78% fat), which gives context for the percentages of fat used in our rodent studies.

### 3.3. Binge-Type Intake and NAc NMUR2 Protein Expression

**Figure 3:**
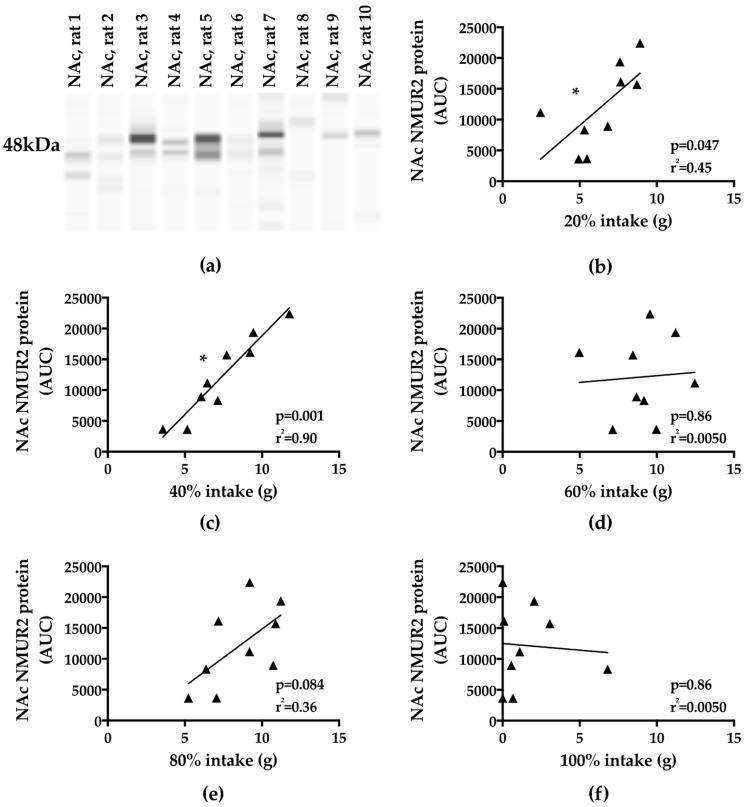
NAc NMUR2 protein expression was positively correlated with binge-type eating. (**a**) representative Western blot image for NMUR2 synaptosomal protein from NAc samples; (**b**) NAc NMUR2 expression was positively correlated with intake of a 20% fat (80% carbohydrate) mixture; (**c**) NAc NMUR2 expression was positively correlated with intake of a 40% fat (60% carbohydrate) mixture; (**e**) NAc NMUR2 expression was not correlated with intake of a 60% fat (40% carbohydrate) mixture; (**f**) NAc NMUR2 expression was not correlated with intake of an 80% fat (20% carbohydrate) mixture; (**g**) NAc NMUR2 protein expression was not correlated with intake of a 100% fat (0% carbohydrate) mixture. AUC: area under the curve. * *p* < 0.05.

To determine the relationship between binge-type intake and NMUR2 expression in the NAc, we quantified NAc NMUR2 synaptosomal protein (Figure 3a), and correlated it to fat intake during the binge period (Figure 3b–f). Interestingly, NAc NMUR2 expression demonstrated a strong positive correlation with binge intake of a “lower”-fat (higher carbohydrate) mixture, specifically 20% fat (80% carbohydrate) (*p* = 0.047) and 40% fat (60% carbohydrate) (*p* < 0.001) (Figure 3b–c). As fat content increased and carbohydrate content decreased, the relationship diminished. However, no significant correlation was observed when NAc NMUR2 was correlated to total calories consumed to account for energy density. In addition, we found no significant correlation between NAc NMUR2 and binge intake of a 60% (40%) (*p* = 0.86), 80% (20%) (*p* = 0.084), or 100% (0%) (*p* = 0.86) fat (carbohydrate) mixture (Figure 3d–f).

### 3.4. Binge-Type Intake and VTA NMUR2 Protein Expression

**Figure 4:**
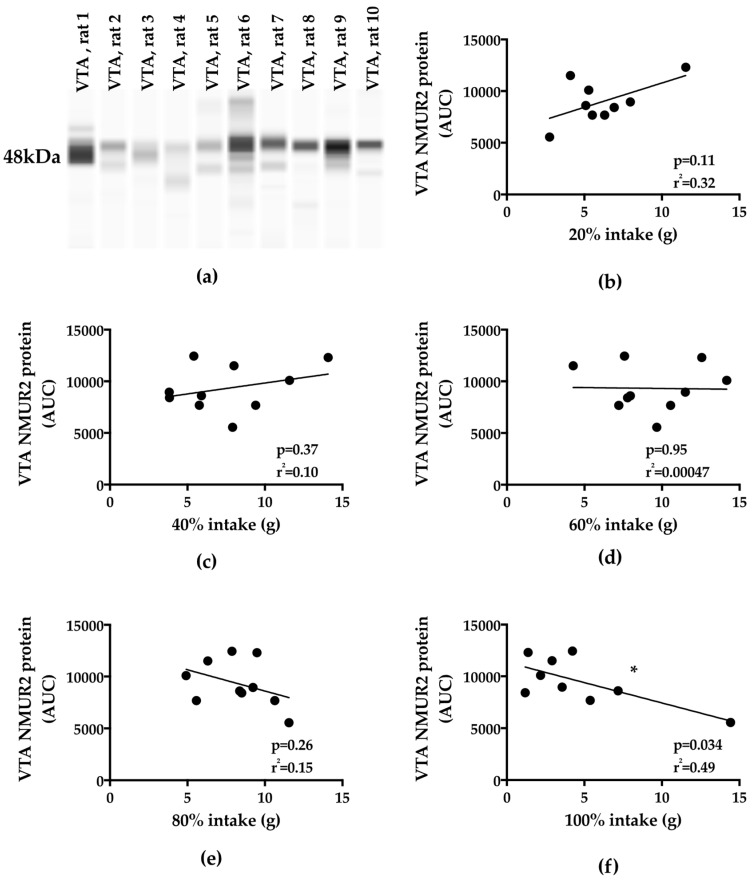
VTA NMUR2 protein expression was negatively correlated with binge-type eating. (**a**) representative Western blot image for NMUR2 synaptosomal protein from VTA samples; (**b**) VTA NMUR2 expression was not correlated with intake of a 20% fat (80% carbohydrate) mixture; (**c**) VTA NMUR2 expression was not correlated with intake of a 40% fat (60% carbohydrate) mixture; (**d**) VTA NMUR2 expression was not correlated with intake of a 60% fat (40% carbohydrate) mixture; (**e**) VTA NMUR2 expression was not correlated with intake of an 80% fat (20% carbohydrate) mixture; (**f**) VTA NMUR2 expression was negatively correlated with intake of a 100% fat (0% carbohydrate) mixture. * *p* < 0.05.

Next, we quantified VTA NMUR2 synaptosomal protein (Figure 4a). We correlated VTA NMUR2 synaptosomal protein to fat intake during the binge period (Figure 4b–f). No significant correlation was observed between VTA NMUR2 and binge intake of a 20% (80%) (*p* = 0.11), 40% (60%) (*p* = 0.37), 60% (40%) (*p* = 0.95), or 80% (20%) (*p* = 0.26) fat (carbohydrate) mixture (Figure 4b–e). VTA NMUR2 demonstrated a strong negative correlation with binge intake of an “extreme” high-fat mixture of 100% fat (0% carbohydrate) (*p* = 0.034) (Figure 4f). Interestingly, the slope of the trend line transitioned from a positive slope value for the lower-fat mixtures (higher carbohydrate) to a negative slope for the higher-fat mixtures (lower carbohydrate).

## 4. Discussion

In the present study, we assessed the synaptic localization of NMUR2 in the NAc and the VTA. Our results support previous studies indicating synaptic NMUR2 expression in the NAc. Additionally, these results extend our knowledge by demonstrating Western blot staining of NMUR2 in synaptosomal VTA fractions, and by visualizing punctate staining patterns for NMUR2 in the VTA, consistent with synaptic localization [46]. Our NMUR2 antibody has previously been shown to stain NMUR2-expressing cell bodies in the PVN, suggesting that the staining pattern is not an antibody limitation [32]. In the NAc, NMUR2 is localized to synapses that express a neuronal marker of GABAergic signaling, which supports a role for NMUR2 in inhibitory signaling [46]. Additional studies are needed to determine the functional role of NMUR2 in the mesoaccumbens circuit, including its effects on BED and obesity.

We used an animal model of binge-type eating to quantify binge intake in rats. This model only simulated a binge episode, similar to that observed in BED and obesity [27,29,30,32,33,35]. One of the major limitations of our study is the inability of our model to replicate all clinical aspects of BED. While our model can quantify food intake during a binge episode, it does not replicate the psychological aspects of BED, such as feelings of loss of control and feelings of shame and guilt [2,3,4,5]. Nonetheless, this model is helpful in understanding neural mechanisms underlying binge-type consumption and identifying potential regulators of this specific overconsumption behavior. During our studies, animals were maintained on their normal chow consisting of 17% fat by kcal which does not promote overconsumption and maintains a healthy energy balance. However, energy-dense diets are reinforcing and are significantly preferred by rodents. The fat–carbohydrate mixtures were designed to contain the preferred higher energy density, which promotes overeating and improves the translational relevance in which humans binge on energy-dense foods [13,35,51,52,53]. 

Using this model, we quantified binge-type consumption across a spectrum of fat–carbohydrate mixtures and correlated it to synaptosomal NMUR2 protein expression in the NAc and the VTA in rats. To our knowledge, we are the first to quantify synaptosomal NMUR2 protein in the NAc and VTA and the first to explore relationships between NMUR2 expression and binge intake. Our results demonstrated a strong positive correlation between synaptosomal NMUR2 protein expression in the NAc and binge intake of a “lower”-fat (higher carbohydrate) mixture of 20% fat (80% carbohydrate) and 40% fat (60% carbohydrate). This region-specific effect of NAc NMUR2 facilitated the binge-type intake of a “lower”-fat (higher carbohydrate) mixture, especially food around 40% fat (60% carbohydrate), which is commonly overconsumed in humans [13,51,52,53,54]. Synaptosomal VTA NMUR2 protein expression demonstrated a strong negative correlation with the binge intake of an “extreme” high fat mixture, particularly 100% fat (0% carbohydrate). Contrary to NAc NMUR2, this finding implicates VTA NMUR2 as an inhibitor of binge intake of “extreme” high-fat, low-carbohydrate food. Thus, VTA NMUR2 may act as a “behavioral brake” on the binge intake of high-fat, low-carbohydrate food.

Overall, our findings demonstrate key relationships and brain-region-specific differences in synaptosomal NMUR2 protein expression, and establish NMUR2 as a regulator of binge-type intake in rats in the NAc and the VTA. Binge-type eating changes based on fat–carbohydrate content and NMUR2 expression varies not only across animals, but across brain regions. Interestingly, binge-type intake could be based on fat content, carbohydrate content, or fat-to-carbohydrate ratio, and future studies should delineate the role of NMUR2 in fat and carbohydrate preference. Thus, endogenous NMUR2 may be a driver of individual differences in binge-type eating. Although the observed high degree of inter-animal variability and inter-brain region variability of NMUR2 can limit data interpretation based on averaged differences in NMUR2, it also raises the possibility that NMUR2 protein contributes to individual differences for binge-type food intake in humans. 

The clinical implications of our findings establish NMUR2 as a novel regulator of binge-type eating and therefore as a potential druggable candidate for the overconsumption behavior observed in BED and obesity. Recently, we showed that small-molecule NMUR2 agonists successfully suppressed high-fat food intake in rats, which supports NMUR2 as a viable therapeutic target [55]. Future studies will continue to investigate the contribution of NMUR2 in the NAc and the VTA at molecular, neural, and pharmacological levels, including investigating the effects of these small-molecule NMUR2 agonists on binge-type eating.

## 5. Conclusions

The overconsumption behavior observed in BED and obesity is poorly understood, and demands more mechanistic and molecular studies. NMUR2 facilitates binge-type eating in rats by promoting the binge intake of “lower”-fat diets via the NAc while suppressing the intake of “extreme” higher fat diets via the VTA. Therefore, NMUR2 represents a promising druggable target, and has already been shown to successfully alter feeding behavior [55,56,57]. The current study demonstrates key relationships between NMUR2 expression in “reward” centers of the brain and binge-type behavior that will contextualize the interpretation of future research determining the therapeutic potential of NMUR2 to regulate overconsumption behavior.
